# Muscle-Invasive Bladder Cancer in Non-Curative Patients: A Study on Survival and Palliative Care Needs

**DOI:** 10.3390/cancers16193330

**Published:** 2024-09-29

**Authors:** Félix Guerrero-Ramos, Daniel Antonio González-Padilla, Santiago Pérez-Cadavid, Esther García-Rojo, Ángel Tejido-Sánchez, Mario Hernández-Arroyo, Carmen Gómez-Cañizo, Alfredo Rodríguez-Antolín

**Affiliations:** 1 Department of Urology, Hospital Universitario 12 de Octubre, Avenida de Córdoba s/n, 28041 Madrid, Spain; 2 Department of Urology, Clínica Universidad de Navarra, 28027 Madrid, Spain; 3 Department of Urology, Hospital Universitario de Ciudad Real, 13005 Ciudad Real, Spain; 4 Department of Urology, HM Hospitales, 28015 Madrid, Spain

**Keywords:** bladder cancer, survival, supportive care, prognosis

## Abstract

**Simple Summary:**

This study investigates the survival outcomes and palliative care needs of patients with muscle-invasive bladder cancer (MIBC) who are not eligible for curative treatment. Analyzing a cohort of 142 patients, this research reveals a median overall survival of 10.6 months and a median cancer-specific survival of 11.9 months. Worse outcomes were associated with advanced disease stage and hydronephrosis. Notably, patients excluded from curative treatment solely due to advanced age had a relatively better prognosis compared to those with severe comorbidities. This study underlines the significant burden on this patient population, highlighting frequent emergency department visits and the need for palliative interventions. These findings emphasize the critical unmet need for tailored therapeutic approaches in patients with MIBC who cannot undergo curative treatment.

**Abstract:**

Objective: To assess the survival outcomes of patients diagnosed with muscle-invasive bladder cancer (MIBC) who are not candidates for curative treatment and to identify the factors influencing these outcomes. Methods: We conducted an analysis of patients diagnosed with MIBC who were either unable or unwilling to undergo curative therapy. We evaluated overall survival (OS) and cancer-specific survival (CSS) and examined their associations with various clinical variables. Additionally, we assessed emergency department visits and palliative procedures. Results: The study included 142 patients with a median age of 79.4 years and a Charlson Comorbidity Index of 9.8. At diagnosis, 59.2% of the patients had localized disease, 23.2% had metastatic disease, and 49.3% presented with hydronephrosis. Curative treatment was excluded due to comorbidities in 40.1% of cases and advanced disease stage in 36.6%. The 1-year and 2-year OS rates were 42.8% and 23.6%, respectively, with a median survival of 10.6 months. The 1-year and 2-year CSS rates were 49.6% and 30.2%, respectively, with a median survival of 11.9 months. Worse survival outcomes were associated with advanced disease stage and the presence of hydronephrosis. Patients excluded from curative treatment solely due to age had a relatively better prognosis. On average, patients visited the emergency department three times: 19% underwent palliative transurethral resection of the bladder tumor, 14.8% received radiotherapy to control hematuria, and nephrostomy tubes were placed in 26.1% of cases. Conclusions: Patients with MIBC who are unable or unwilling to undergo curative treatment have a median overall survival of less than one year, with worse outcomes observed in those with advanced disease stage and hydronephrosis.

## 1. Introduction

Bladder cancer is the seventh most common cancer in men and the seventeenth most common in women worldwide, according to the GLOBOCAN project. Spain is among the countries with the highest rates of bladder cancer, where it ranks as the fifth most common cancer in terms of incidence and fourth in prevalence. The top three countries with the highest incidence of bladder cancer are Belgium, Lebanon, and Malta [1,2]. It is estimated that approximately 75% of patients are diagnosed with non-muscle-invasive bladder cancer (NMIBC), while the remaining 25% present with muscle-invasive bladder cancer (MIBC) [1,3]. Among patients with NMIBC, it is estimated that approximately 7.1% to 19.8% will progress to MIBC within five years, regardless of the therapies administered and depending on the initial stage and grade at diagnosis [4,5,6]. Despite significant efforts to develop new alternatives for NMIBC patients to reduce recurrence and progression rates, a substantial number of patients still experience disease progression to MIBC, which poses a serious threat to their survival [7,8].

The standard surgical treatment for non-metastatic MIBC is neoadjuvant cisplatin-based chemotherapy (in eligible individuals), followed by radical cystectomy with urinary diversion. Other curative alternatives include partial cystectomy (in highly selected cases) or multimodal bladder-preserving strategies that combine chemotherapy and radiotherapy [9]. For patients in whom curative intent is pursued, numerous new alternatives are being investigated, including novel drugs in adjuvant or neoadjuvant settings, as well as the potential need for critical care therapy [10,11,12,13,14,15]. Conversely, patients deemed unsuitable for curative treatment have limited options to improve their survival, highlighting the unmet needs of this population.

When comparing younger individuals with octogenarians, age has not been shown to be a decisive factor in determining treatment modalities. Carefully selected patients in both age groups have demonstrated similar outcomes following radical cystectomy [16,17]. However, some patients cannot be offered curative treatment, typically due to serious comorbidities, advanced age, or a metastatic stage at diagnosis. Managing this group of patients presents a significant challenge in daily clinical practice, particularly when it comes to communicating the prognosis and estimated survival to patients and their families.

In recent years, there has been a remarkable increase in the number of patients with advanced age and multiple comorbidities, a trend largely driven by the progressive increase in life expectancy. As people are living longer, healthcare systems are experiencing more complex cases where patients present with a combination of advanced age and chronic health conditions, making them more vulnerable to serious diseases like cancer. In Spain, for instance, life expectancy has risen significantly, from 77 years in 1990 to 83.2 years in 2019 [18]. As a result of this aging population, many patients are no longer considered suitable candidates for curative treatment, either due to the risks associated with their comorbidities or because the potential benefits of such aggressive treatments are outweighed by the challenges posed by their overall health. This has created an urgent need to better understand the prognosis of these patients, who are often excluded from curative treatment options. Gaining a deeper understanding of their expected outcomes is crucial, as it would enable healthcare providers to offer more accurate and personalized information to these patients and their families. In doing so, clinicians can help patients and caregivers make more informed decisions about care and treatment planning while also setting realistic expectations about what can be achieved in terms of survival and quality of life.

Several studies have shown that the majority of patients with advanced-stage cancer express a desire to know their prognosis and expected survival, as this information can help them make well-founded decisions regarding their treatment and future plans. However, it is also important to recognize that not all patients share the same preferences when it comes to receiving such sensitive information. Some individuals prefer to be informed of only the most essential details, choosing to avoid full disclosure of their prognosis in order to focus on preserving hope or emotional well-being. Additionally, a smaller subset of patients may opt not to be informed at all, finding the information too overwhelming or distressing to the process. This diversity in preferences highlights the importance of personalized communication strategies, wherein healthcare providers must carefully assess each patient’s readiness and willingness to receive detailed information regarding their prognosis [19,20].

Given the scarcity of data in the literature regarding the survival outcomes of patients with MIBC who are not eligible for curative treatment, we decided to conduct an analysis of survival outcomes for this specific patient group at our center. We aimed to determine overall survival (OS) and cancer-specific survival (CSS), providing a clearer understanding of their prognosis in the absence of curative interventions. In addition, we sought to identify and analyze the various factors that may influence survival in this population, including demographic, clinical, and treatment-related variables; these factors can help us better tailor care and improve prognostication. We also evaluated the frequency with which these patients required emergency department visits, as this often reflects disease complications or unmet care needs. Lastly, we assessed the necessity of palliative procedures related to their disease, such as interventions aimed at managing symptoms or complications, to gain insight into the palliative care needs of this vulnerable group.

## 2. Methods

We conducted a retrospective review of electronic health records for patients diagnosed with MIBC following transurethral resection of a bladder tumor (TURBT) at our Tertiary Referral Academic Center over a five-year period. The inclusion criteria for the study encompassed every patient diagnosed with MIBC, regardless of stage, as long as a curative treatment approach had been deemed unsuitable. This was intended to capture a wide range of patients who, for various reasons—such as advanced disease, comorbidities, or other limiting factors—were not considered candidates for potentially curative interventions like surgery, radiotherapy, or chemotherapy. In contrast, the exclusion criteria ensured the accuracy of diagnosis and relevance of the study population. Patients who had only been diagnosed with MIBC through imaging techniques, without histopathological confirmation of the disease, were excluded to prevent misclassification. Furthermore, any individuals who had received any form of curative treatment—whether radical cystectomy, radiotherapy, or other therapies—were also excluded, as the study focused on those for whom curative treatment options were no longer viable. This ensured that the study findings would be directly applicable to this population with limited treatment options.

Data collection was conducted through a comprehensive review of the patients’ electronic health records. Basic demographic information, such as age, sex, and co-existing medical conditions, was collected alongside clinicopathological variables. These included the date of MIBC diagnosis, distinction between urothelial and non-urothelial histological subtypes, and comorbidities present at the time of diagnosis, which were assessed using the Charlson Comorbidity Index (CCI) to quantify the overall disease burden. In addition, we recorded the clinical tumor-node-metastasis (TNM) staging, as determined by imaging studies, which was then simplified into three categories: localized disease, lymph node involvement (N1–N3), or metastatic disease (M1a–M1b). We also documented whether the muscle-invasive bladder tumor was a primary diagnosis or represented progression from NMIBC. Finally, the presence or absence of hydronephrosis at the time of diagnosis was recorded, given its potential prognostic role.

To identify the reasons for ruling out curative treatment, each decision was categorized into one of four groups: comorbidities, advanced stage (metastatic or nodal disease), advanced age (over 80 years), or patient refusal of treatment. Additionally, we recorded the number of visits to the emergency department related to the bladder tumor diagnosis, as well as the need for palliative procedures such as TURBT, radiotherapy, percutaneous nephrostomy, and embolization of the hypogastric arteries.

Survival outcomes were estimated using Kaplan-Meier curves. To compare survival rates between different groups, we employed the LogRank test. To explore the relationship between various clinicopathological and demographic variables and patient survival, we utilized the Cox proportional hazards regression model. This model was applied to both univariate and multivariate analyses, enabling us to examine the independent effect of each variable on survival outcomes. Through univariate analysis, we assessed the individual impact of each factor, while the multivariate approach allowed for the evaluation of multiple variables simultaneously, adjusting for potential confounders. For all statistical analyses, significance was determined by a *p*-value of less than 0.05. Data analysis was conducted using Stata (StataCorp©, College Station, TX, USA) version 12 for Windows.

## 3. Results

Among the 142 patients included in the study, the median age was 79.4 years, with 87.3% of the sample being male and a median CCI of 9.8. Regarding tumor characteristics, 61.2% of the patients were diagnosed with MIBC without a prior history of bladder cancer (primary MIBC diagnosis). More than half of the tumors (59.2%) were classified as localized disease, 17.6% presented with lymph node involvement without distant metastases, and 23.2% had radiological evidence of distant metastases. Among the patients with distant metastases, the majority presented with either bone (15 patients) or lung (9 patients) involvement. Other sites of secondary deposits included the liver (4 patients), liver and lung (2 patients), liver and bone (2 patients), and lung and bone (1 patient).

Forty percent of patients were excluded from curative treatment due to comorbidities, one-third due to advanced-stage disease, and one in five due to advanced age. A multidisciplinary tumor board made the decision to forgo curative intent due to comorbidities. This decision considered not only the individual chronic conditions (such as diabetes mellitus, chronic kidney disease, heart failure, etc.) but also the cumulative impact of these comorbidities on the patients’ overall performance status. The majority of patients in this series (61.9%) were managed with observation alone, without any initial palliative interventions offered for their disease. Table 1 provides a summary of the most relevant baseline characteristics of our cohort.

**Figure 1:**
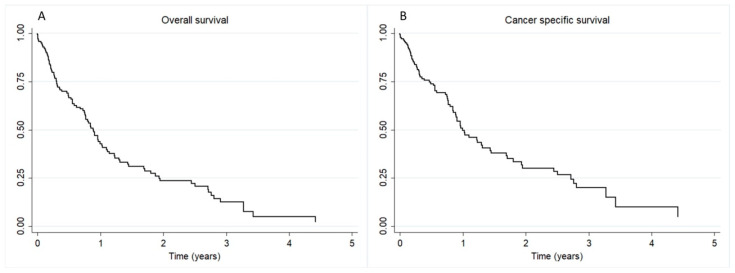
Survival curves.

The median OS and CSS were 10.6 months and 11.9 months, respectively. These estimates are illustrated in Figure 1. The one year OS rate was 42.8%, declining to 23.6% in two years. CSS rates were 49.6% and 30.2% at one and two years, respectively.

From the time of diagnosis and the decision not to pursue curative treatment until their death, patients visited the emergency department an average of three times due to bladder cancer complications, with a mean hospitalization length of 3.4 days. During this period, 27 patients (19%) required palliative TURBT to manage hematuria, 21 patients (14.8%) underwent palliative radiotherapy for the same reason (three sessions of 7 Gy administered on alternate days), 37 patients (26.1%) needed urinary diversion via a percutaneous nephrostomy catheter, and four patients (2.8%) were treated with embolization of the hypogastric arteries due to persistent hematuria (after failure of TURBT, radiotherapy, or both). Once the diagnosis is confirmed and the decision not to pursue curative intent was made, all of our patients were referred to the Palliative Care Unit for management and close follow-up.

**Figure 2:**
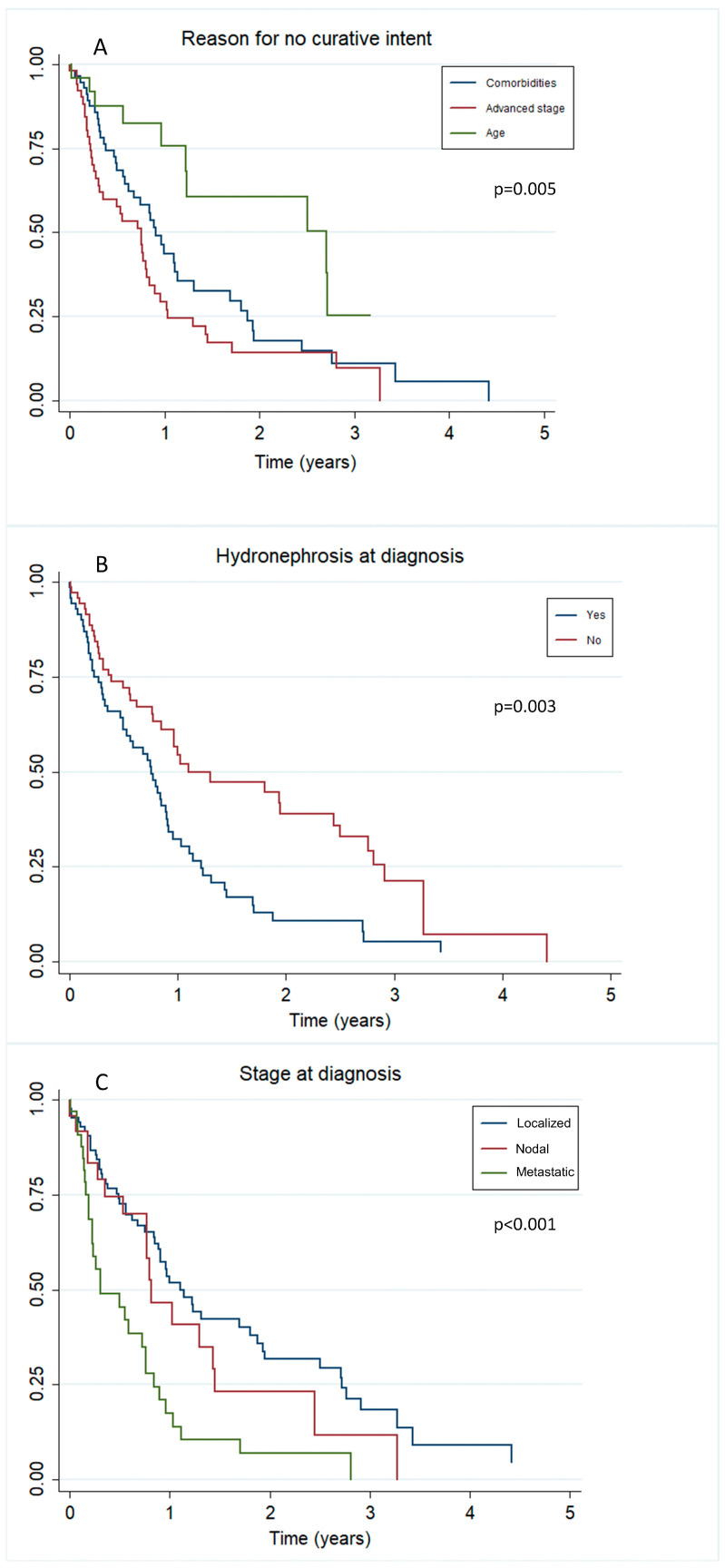
Stratification of overall survival based on the variables related to prognosis: (**A**) reason for deciding no curative intent, (**B**) hydronephrosis at diagnosis, and (**C**) stage at diagnosis.

Univariate analysis identified several factors as predictors of worse overall survival, including the presence of metastatic disease, hydronephrosis at diagnosis, a CCI score of 9 or higher, and advanced age as the reason for being deemed unsuitable for curative treatment. Following multivariate analysis, the presence of metastases and hydronephrosis at diagnosis emerged as the most robust independent predictors of poor overall survival. Specifically, patients with metastases had a hazard ratio (HR) of 2.06, while those with hydronephrosis had a HR of 1.91, indicating a significantly higher risk of mortality in these groups. Patients excluded from curative treatment due to advanced age demonstrated better survival outcomes compared to those excluded due to comorbidities, with an HR of 0.49 (as shown in Table 2 and Figure 2). Although metastases were identified as predictors of poor outcomes, our analysis did not reveal any significant survival differences based on the location of the metastatic sites.

## 4. Discussion

Our study highlights a critical issue in this underrepresented and underserved population, showing a median overall survival of less than one year. Furthermore, after four years, fewer than 25% of patients remain alive, highlighting the aggressive nature of this stage of the disease and the limited long-term survival expectations for these individuals. This information provides valuable insights into a question frequently encountered in daily clinical practice, where clinicians often face uncertainty due to the paucity of comprehensive studies focusing on this particular population. Additionally, the minimal difference observed between OS and CSS at both the one- and two-year marks strongly indicates that bladder cancer is the primary cause of death for the majority of these patients. This suggests that competing risks from other health conditions play a less significant role in mortality, further emphasizing the lethal impact of bladder cancer in this group.

Two papers have reported on similar populations, both focusing on Swedish patients [21,22]. One study reported a median overall survival of 9 months, while the other reported a median OS of 8 months. Additionally, a more recent study involving Japanese patients reported a slightly longer median OS of 12 months [23]. The publication of these studies, along with our findings, underlines the existence of patients with MIBC who do not receive curative treatment after TURBT. This body of evidence stresses the pressing need to better understand this specific group of subjects, as they represent a population with significant unmet medical needs, accentuating the constraints for future research and therapeutic advancements in this area.

While a significant increase in OS has been observed in patients with metastatic bladder cancer following the approval of several new therapies across both first- and subsequent-line treatments, those who are unfit for curative treatment continue to face a poor prognosis. Unfortunately, there have been no substantial advancements in this population [24,25,26,27,28,29]. In this regard, only one phase I trial has been published to date on the use of TAR-200 in these individuals [30]. TAR-200 is a novel investigational drug delivery system designed for the localized treatment of bladder cancer. It consists of a small, flexible intravesical device that is inserted into the bladder through a urethral catheter, slowly and continuously releasing gemcitabine over a three-week period. In this phase I trial, the authors reported results from a cohort of 35 patients and achieved an overall response rate of 40.0%. The median overall survival was 27.3 months, and the median duration of response was 14.0 months. Notably, over two-thirds of the patients (70.5%) remained progression-free at 12 months, suggesting that TAR-200 could offer meaningful benefits for this underserved patient group.

Our data also draw attention to the significant disease burden caused by MIBC when curative treatment is not an option. Hematuria and other bladder cancer-related issues frequently lead patients to seek treatment at our facilities throughout the course of their illness. Consequently, there is a high incidence of invasive palliative procedures, which can substantially reduce the quality of life due to frequent hospital admissions and complications associated with these interventions [31]. A similar disease burden has been reported in previous studies, reinforcing the challenges faced by this patient population. These studies also highlight the frequent need for palliative interventions and their associated impact on quality of life, which is consistent with our findings [22]. These findings will be crucial when informing patients and their families about the expected progression of the disease following diagnosis and the decision not to pursue curative treatment. Providing this information can help set realistic expectations and guide discussions about care plans and quality-of-life considerations. Future research could explore patient-centered communication frameworks that help clinicians provide prognosis information while respecting patient preferences. Studies focusing on shared decision-making models may improve the satisfaction and emotional well-being of patients and their families.

Some centers consider radiotherapy alone for these patients after diagnosis, and once curative intent has been ruled out. However, in our center, each patient is discussed during our weekly multidisciplinary tumor board meetings. We typically do not opt for radiotherapy alone upfront, as there is no evidence of improved survival, which can lead to significant toxicity in some cases. Instead, we reserve radiotherapy for select individuals when managing refractory hematuria or other complications.

Consistent with previously published data, our study shows poorer survival outcomes for patients with metastatic disease compared to those with localized tumors. Additionally, our multivariate analysis indicates that hydronephrosis at diagnosis is an adverse prognostic factor. While this has not been previously demonstrated in studies involving patients unsuitable for curative therapy, it has been established as an independent risk factor in patients undergoing cystectomy [32,33]. These findings could prompt us to consider initiating the best supportive care earlier for this subgroup of patients, given their reduced chances of survival. Early intervention with supportive care may help improve the quality of life and better manage symptoms in this high-risk population. Moreover, given the significant palliative needs observed in these individuals, future research should focus on improving palliative care strategies, such as more efficient management of hematuria or pain in patients with MIBC. Investigating new palliative interventions, including less invasive procedures or novel local treatments, may help enhance the quality of life of these patients. Additionally, studies exploring the optimal timing and type of supportive care that produces the best outcomes would be highly valuable.

The reasons for ruling out curative treatment have not been addressed in previous studies. However, our data indicate that patients for whom curative therapy was dismissed due to advanced age have a better prognosis than those excluded due to comorbidities. Moreover, age alone does not appear to be a limiting factor for overall survival in our series, which aligns with the published literature on cystectomy in octogenarian patients [16,17]. This conclusion is in line with findings from other groups, where a multicenter prospective study concluded that the risk-benefit balance of radical surgery improves significantly with careful patient selection [34].

We acknowledge several limitations of our study, one of which is its retrospective nature. However, we believe that the retrospective design does not significantly impact our results, as a large proportion of the patients included in this survival analysis reached the primary endpoint (death).

Although the majority of subjects were managed with observation alone, some received palliative treatments, such as radiotherapy for hematuria and chemotherapy with suboptimal regimens and doses. This may have introduced some degree of heterogeneity into the series, which in practical terms reflects real-world clinical practice. Consequently, this may enhance the external validity of our findings, particularly given the inclusion of patients with varying disease stages within our population. However, since these treatments were not intended to be curative, they likely did not impact survival outcomes. Instead, they may have improved the patient’s quality of life. Immune checkpoint inhibitors are currently an option for this population, including those at our center. However, this cohort reflects the period before reimbursement was available in our country; therefore, no patient in this study received this therapy. This may have introduced some bias into our results.

Despite being a single-center cohort, our results are consistent with those of previous population-based studies, indicating that this condition is both widespread and underreported. The similarity of outcomes across different settings emphasizes the strength and applicability of our findings [21,22]. In this regard, future research should focus on conducting comparative studies across various geographical regions to help validate the findings and determine whether cultural, socioeconomic, or healthcare system factors influence survival outcomes and care needs in patients with non-curative MIBC. Such studies could provide valuable insights into how differences in healthcare access, treatment protocols, and patient support systems across diverse populations impact prognosis and quality of life. Understanding these variations may ultimately guide the development of more customized interventions and inform global best practices for managing patients with MIBC who are ineligible for curative treatment.

Finally, our findings highlight the importance of addressing the needs of this overlooked population, particularly patients with localized muscle-invasive disease who are not ideal candidates for curative therapy. The development of new agents or devices for this condition could provide valuable options for physicians who often face the challenging decision between pursuing curative treatment and opting for palliative care in these cases. Therefore, there is a need to explore the role of emerging therapies (such as immune checkpoint inhibitors or novel drug delivery systems like TAR-200) specifically in non-curative populations. Trials focusing on quality of life as an endpoint could provide more patient-centered approaches.

## 5. Conclusions

The median survival for patients with MIBC who are not eligible for curative treatment is less than one year, during which they often require frequent emergency department visits and undergo numerous palliative invasive procedures. Factors associated with poorer prognosis include metastatic stage and the presence of hydronephrosis at diagnosis. In contrast, patients for whom curative treatment is ruled out solely due to advanced age tend to have a better prognosis, indicating that age alone should not be considered a contraindication for radical cystectomy.

This information is crucial for clinicians to provide more informed guidance to patients and their families and highlights the significant unmet needs within this patient population.

## Figures and Tables

**Table 1:** Baseline characteristics.

	*n* = 142
Age, median (±SD)	79.4 (±7.8)
Sex, *n* (%) male	124 (87.3)
Charlson comorbidity index, Median (±SD)	9.8 (±7.1)
New diagnosis, *n* (%)	88 (61.2)
Histology, *n* (%)	
Urothelial	134 (94.4)
Epidermoid	8 (5.6)
Clinical stage at diagnosis, *n* (%)	
Localized	84 (59.2)
Nodal (N1–N3)	25 (17.6)
Metastatic (M1a–M1b)	33 (23.2)
Hydronephrosis at diagnosis, *n* (%)	70 (49.3)
Reason for no curative intent, *n* (%)	
Comorbidities	57 (40.1)
Advanced clinical stage	52 (36.6)
Age	25 (17.6)
Patient/relatives’ refusal	6 (4.2)
Unknown	2 (1.5)
Treatment offered, *n* (%)	
Observation	88 (61.9)
Palliative radiotherapy	20 (14.1)
Cutaneous ureterostomy	6 (4.2)
Transurethral resection of bladder tumor	4 (2.8)
Palliative chemotherapy (carboplatin-gemcitabine or gemcitabine alone)	17 (12.0)
Palliative chemo-radiotherapy	7 (5.0)

**Table 2:** Univariate and multivariate analyses for the risk of death from any cause (HR: hazard ratio, CI: confidence interval). In bold, statistically significant variables.

Variable	Univariate Analysis		Multivariate Analysis	
	HR (CI 95%)	*p*	HR (CI 95%)	*p*
Male sex	0.69 (0.37–1.28)	0.25	0.61 (0.33–1.14)	0.12
Stage				
Nodal (N1–N3) vs. Localized	1.39 (0.80–2.42)	0.24	1.46 (0.84–2.58)	0.18
Metastatic (M1a–M1b) vs. Localized	2.73 (1.71–4.35)	**<0.001**	2.06 (1.09–3.86)	**0.025**
Hydronephrosis at diagnosis	1.83 (1.22–2.75)	**0.003**	1.91 (1.26–2.89)	**0.002**
Reason for no curative intent				
Stage vs. Comorbidities	1.45 (0.94–2.24)	0.09	1.17 (0.54–2.59)	0.68
Age vs. Comorbidities	0.43 (0.21–0.86)	**0.017**	0.49 (0.21–0.97)	**0.042**
Age, ≥80	0.79 (0.53–1.19)	0.26	0.82 (0.53–1.26)	0.37
Charlson comorbidity index, ≥9	1.77 (1.18–2.65)	**0.005**	1.35 (0.78–2.32)	0.28

## Data Availability

The data that support the findings of this study are available from the corresponding author upon reasonable request.
